# Two-Dimensional Nanomaterials for Gas Sensing Applications: The Role of Theoretical Calculations

**DOI:** 10.3390/nano8100851

**Published:** 2018-10-19

**Authors:** Yamei Zeng, Shiwei Lin, Ding Gu, Xiaogan Li

**Affiliations:** 1 State Key Laboratory of Marine Resource Utilization in South China Sea, Hainan University, Haikou 570228, China; zengyamei00@163.com; 2 School of Electronic Science and Technology, Institute for Sensing Technology, Dalian University of Technology, Dalian 116024, China; guding815@mail.dlut.edu.cn

**Keywords:** 2D nanomaterials, gas sensing, theoretical calculations

## Abstract

Two-dimensional (2D) nanomaterials have attracted a large amount of attention regarding gas sensing applications, because of their high surface-to-volume ratio and unique chemical or physical gas adsorption capabilities. As an important research method, theoretical calculations have been massively applied in predicting the potentially excellent gas sensing properties of these 2D nanomaterials. In this review, we discuss the contributions of theoretical calculations in the study of the gas sensing properties of 2D nanomaterials. Firstly, we elaborate on the gas sensing mechanisms of 2D layered nanomaterials, such as the traditional charge transfer mechanism, and a standard for distinguishing between physical and chemical adsorption, from the perspective of theoretical calculations. Then, we describe how to conduct a theoretical analysis to explain or predict the gas sensing properties of 2D nanomaterials. Thirdly, we discuss three important methods that have been applied in order to improve the gas sensing properties, that is, defect functionalization (vacancy, edge, grain boundary, and doping), heterojunctions, and electric fields. Among these strategies, theoretical calculations play a very important role in explaining the mechanisms underlying the enhanced gas sensing properties. Finally, we summarize both the advantages and limitations of the theoretical calculations, and present perspectives for further research on the 2D nanomaterials-based gas sensors.

## 1. Introduction

Gas sensors are devices used for detecting the composition and concentration of gases, which are applied in many fields, including in resident life, industry, military, and medical treatment [1,2]. As a key component, sensing materials play a critical role in the performance of gas sensors. Various materials have been investigated for gas sensing, including semiconducting metal oxides [3,4,5], conducting polymers [6], carbon nanotubes (CNTs) [7,8,9], and so on [10]. Among these sensing materials, metal oxides have been extensively investigated in the past few decades, because of their high sensitivity and low cost [11]. However, a high operating temperature (OT) [12] and low selectivity have been their limits. Recently, there have been some attempts looking at the possibility of using metal oxides at room temperature or nearly room temperature, or using light activation instead of heating [13]. But the underlying mechanism is still not clear now. Although conducting polymers can work at room temperature (RT) and can be easily fabricated, the effects of humidity and the performance degradation still limit the ability of the conducting polymer to become incorporated into sensing devices [14]. As for carbon nanotubes, they possess room temperature operability and a superb sensitivity, however, poor reversibility and complex processing still hinders their practical application [15]. Thus, it is urgent to explore novel gas-sensing materials with a high sensitivity, selectivity, stability, reversibility, and low operating temperatures.

Since the discovery of graphene, a unique two-dimensional layered structure, various 2D nanomaterials have been extensively studied for their use in low temperature gas sensors, both in traditional experiments and in theoretical calculations [16,17]. 2D nanomaterials generally possess a large surface-to-volume ratio, which is advantageous for gas sensing applications as it facilitates surface reactions. In particular, 2D layered nanomaterials have aroused a large amount of interest because of their excellent semiconducting performances, and because they can be easily configured into chemiresistive field effect transistors (FETs). Other attractive features are the unique thickness dependent physical and chemical properties, particularly in transition metal dichalcogenides (TMDs). 

Compared to the traditional experimental methods, theoretical calculations have the obvious advantage of saving costs both in terms of time and effort. Furthermore, the calculation approach allows for identifying the atomistic processes of gas sensing, and for exploring the intrinsic changes inside the sensing materials. Along with the development of computational materials science, the theoretical calculation methods can not only be used to provide the reasonable and profound explanations for the experimental results, but they are helpful for the design of new materials structures and thus new functionalities.

In this review, we mainly focus on the contribution of the theoretical calculations in order to the study of gas-sensing properties of 2D nanomaterials, such as graphene and its derivatives (reduced graphene oxide and graphene oxide), and TMDs. Theoretical studies of the interactions between gas molecules and 2D sensing materials provided both a theoretical basis, as well as design ideas for developing novel gas-sensing materials with an enhanced gas-sensing performance.

## 2. Gas-Sensing Mechanism of 2D Layered Nanomaterials

Various explanations for their gas sensing behavior have been proposed in the literature. On the basis of theoretical calculations, it is believed that the gas species are physically adsorbed on the surface of 2D layered nanomaterials [18], because of the long distance of adsorption, weak adsorption energy, small amount of charge transfer, and almost constant electronic structure. However, those results were obtained with perfect monolayer materials as a model, and thus the inert surface shows a low sensitivity. As a matter of fact, defects are unavoidably introduced during the material synthesis processes, which are usually more reactive than the perfect lattice sites. Therefore, chemical adsorption between the gas molecules and the defective or functionalized sites can occur on the surface of the 2D nanosheets. Through the density functional theory (DFT) calculations, one can find adsorption distances of gas molecules closer than the sum of two atomic radii. Therefore, the adsorption energy and the amount of charge transfer are much larger compared to the physical adsorption. As a consequence, much larger changes in the electronic properties are induced, caused by the strong hybridization between the gas molecular orbitals and the 2D materials’ orbitals.

Fundamentally, a charge transfer occurs between the gas and the 2D layered nanomaterials, which results in changes in the conductivity or resistivity of the sensing materials [16,19,20]. Two kinds of charge transfer mechanisms are widely proposed [21], namely: (i) A charge transfer that can occur depending on the relative positions of the highest occupied molecular orbital (HOMO) and the lowest un-occupied molecular orbital (LUMO) of the gas molecules. If the HOMO is above the Fermi level of the pure material (the Dirac point), there is a charge transfer to the 2D nanomaterial. If the LUMO is below the Dirac point, the charge will be extracted from the nanosheet towards the gas molecule. (ii) The charge transfer between the gas molecules and 2D nanomaterials is also partially determined by the mixing of the HOMO and LUMO with the 2D nanomaterial orbitals through hybridization. The mixing degree scales with the overlap of the interacting orbitals and the inverse of their energy difference.

When the sensors are exposed to the targeted gas molecules, the gas species are adsorbed on the surface of the 2D sensing materials that are acting as electronic donors or acceptors. Consequently, if the charge affinity of the sensing materials is increased intentionally, a possible improvement of the sensitivity and selectivity of the gas sensors can be expected. Upon exposure to air or nitrogen, the gas molecules are expected to desorb from the surface of the sensing materials. Therefore, hindering the charge transfer is beneficial for the desorption process, leading to an improvement in the reversibility of the gas sensors. The properties of the gas-sensing materials can thus be controlled with respect to the charge affinity of the gas species.

## 3. Theoretical Analysis of Gas-Sensing Properties of 2D Layered Nanomaterials

**Figure 1:**
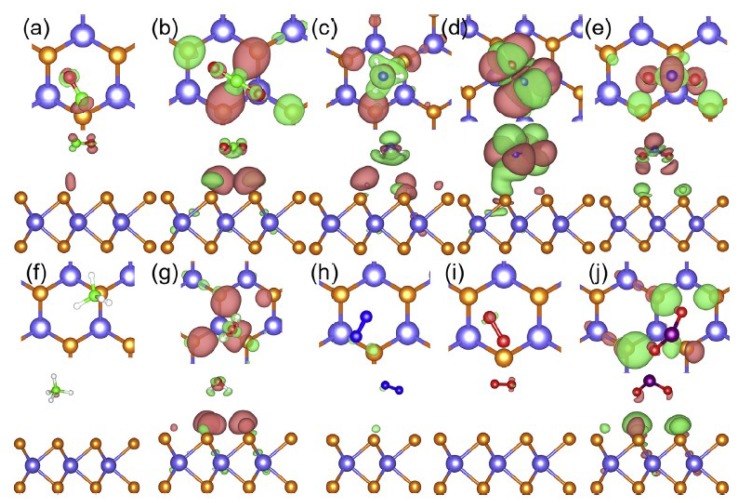
Isosurface plot of the electron charge density difference for (**a**) CO, (**b**) CO_2_, (**c**) NH_3_, (**d**) NO, (**e**) NO_2_, (**f**) CH_4_, (**g**) H_2_O, (**h**) N_2_, (**i**) O_2_, and (**j**) SO_2_ on an MoS_2_ monolayer (top view and side view are provided in the first row and second row for each of the adsorbed molecules). The charge accumulation is represented in pink and the charge depletion is in lime (reproduced from [22], with permission from ELSEVIER, 2018).

**Figure 2:**
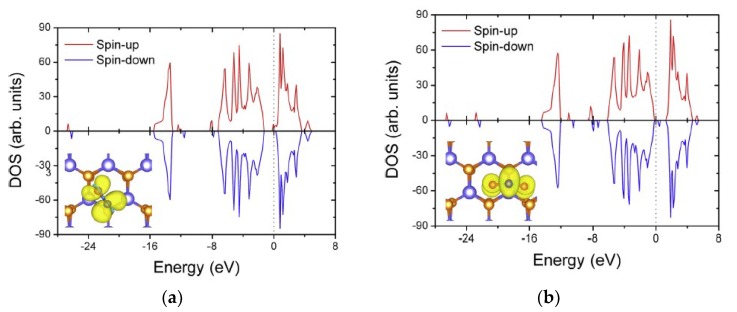
Spin-polarized density of states (DOS) of (**a**) NO and (**b**) NO_2_ adsorbed on MoS_2_ (reproduced from [22], with permission from ELSEVIER, 2018).

The gas-sensing performance of novel materials can be theoretically predicted with the help of DFT calculations. Stable monolayers or several layers of 2D nanosheets are used as a base material for the adsorption of gas molecules. The most stable form of an adsorption model can be determined by means of energy relaxation. Therefore, the positions and orientations of the gas molecules on the surface of the sensing material as well as the distances between them can be easily determined. Furthermore, the interaction of gas molecules on the surface of the sensing materials is explored through the analysis of adsorption energies, energy gap variation, density of states (DOS), electron density, and Bader charges. The adsorption energy confirms the adsorption ability of the gas species. The adsorption energy of the gas molecules on the surface of the sensing materials is defined as follows:

(1) $$\;E_{ad}=E_{gas+material}-E_{matrerial}-E_{gas}\;$$

where $E_{gas+material}\;$ is the energy of the optimized structure of the gas molecule adsorbed on the sensing material, and $E_{material}$ and $E_{gas\;}$, in turn, are the energies of the pristine sensing material layer and of the isolated gas molecule, respectively [22]. Therefore, when $E_{ad}$ attains a negative value, it means that the gas adsorption is a spontaneous and favorable process. The band structure and the DOS give clear insights on the electronic properties of the sensing materials’ nanosheets after gas adsorptions. The Bader charge analysis confirms the charge transfer between the gas molecules and the sensing material. For instance, Figure 1 is an isosurface plot of the electron charge density difference for CO, CO_2_, NH_3_, NO, NO_2_, CH_4_, H_2_O, N_2_, O_2_, and SO_2_ on a MoS_2_ monolayer, and Figure 2 shows the DOS of NO and NO_2_ adsorbed on MoS_2_.

Gas adsorption on most of the 2D layered nanomaterials, such as graphene, MoS_2_, WS_2_, SnS_2_, MoSe_2_, and WSe_2_, has been studied from first-principles calculations. Table 1 shows some typical examples. Although the calculation methods are different, the relative results all exhibit a similar trend. For instance, the adsorption energies of NO_2_ on the WSe_2_, resulting from two different kinds of calculations, both yield the maximal value, which indicates that WSe_2_ possesses the highest sensitivity to NO_2_. However, different algorithms for evaluating the affinity of different target molecules and sensing layers limit the comparison between different gas sensing materials. Therefore, the sensing properties’ comparison of different materials, such as the comparison of WS_2_ and WSe_2_ [23], should be made in a certain gas atmosphere with the same calculation method. From these calculation results, one can judge whether the sensing material is a promising candidate for the chosen gas species or not. For instance, Bui et al. [24] investigated the interaction between a WS_2_ monolayer and several kinds of gas molecules (CO, H_2_O, NO, and O_2_) using first-principle calculations. The reported adsorption energies clearly indicate a physical adsorption between the gas molecules and WS_2_ monolayers. Furthermore, the calculation results show that the NO and O_2_ adsorption narrows the band gap of the material, adding new electronic states in the band gap. Furthermore, according to the Bader charge analysis for all of the structures, NO and O_2_ can withdraw more electronic charge than H_2_O and CO. These observations indicate a promising application of WS_2_ for NO- and O_2_-sensing. In addition, Zhou et al. [25] calculated more gas species (H_2_, NH_3_, and NO_2_) adsorbed on the surface of WS_2_ than mentioned before. According to their paper, monolayer WS_2_ exhibited a better sensitivity with respect to NO_2_ than NO and O_2_, because of the higher adsorption energy and the larger charge transfer. Their theoretical results for the adsorption of NH_3_ and NO_2_ on the WS_2_ monolayers are also consistent with the most recent experimental data [26,27,28]. Li et al. [28] successfully made a room temperature ammonia sensor using WS_2_ nanoflakes as the sensing materials. The sensor showed a p-type response to ammonia. It indicated a good sensitivity and good response/recovery speeds at various concentrations of ammonia from 1 to 10 ppm, as applied at room temperature. The response and recovery times of the sensor to 5 ppm ammonia are ~120 and ~150 s, respectively. Obviously, through the above analysis, the excellent gas sensing materials must possess the following properties: appropriate adsorption energy, a high level of charge transfer, a reduced band gap after the gas molecular adsorption onto the semiconductors.

**Figure 3:**
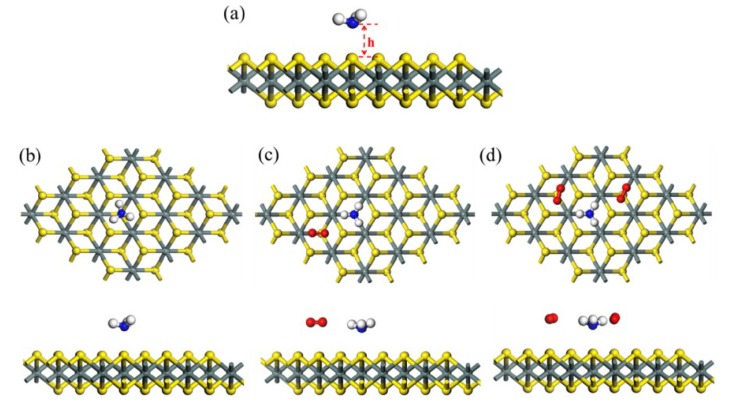
(**a**) Optimized 6 × 6 supercell of SnS_2_ (h represents the closest distance between NH_3_ and the SnS_2_ surface), (**b**–**d**) top views and side views of the SnS_2_ surface with no O_2_, one O_2_, and two pre-adsorbed O_2_ molecules, pre-adsorption. Red, silver, blue, yellow, and cyan balls represent O, H, N, S, and Sn atoms, respectively (reproduced from [29], with permission from ELSEVIER, 2018).

The oxygen and humidity in the air always have a significant impact on the gas detection process. Theoretical calculations can help to understand the influence of oxygen and humidity in the background in gas detection. Xiong et al. [29] calculated the adsorption energy of NH_3_ on the surface of monolayer SnS_2_ with different oxygen concentrations using DFT, as shown in Figure 3. The results showed that the adsorption energies increase with the increase of the oxygen content in the background, indicating that the presence of O_2_ gas promoted the adsorption of NH_3_. According to a previous report, O_2_ usually acts as an electron acceptor, while NH_3_ acts as an electron donor [30]. When the O_2_ molecules adsorb on the surface of SnS_2_ (an n-type semiconductor), there will be a charge transfer from the SnS_2_ to O_2_ molecules, leading to an increase in the resistance of SnS_2_. As a certain concentration of NH_3_ gas is introduced, the adsorbed NH_3_ molecules are expected to donate electrons to SnS_2_, which confirms the decrease in resistance of SnS_2_. In this way, the presence of oxygen enhances the sensor response to ammonia.

## 4. Enhancement of the Gas-Sensing Performance of 2D Layered Nanomaterials

### 4.1. Defect Functionalization

Defects are unavoidably introduced during material synthesis processes. Examples are point defects, dislocations, grain boundaries, and edges, which are usually more reactive than normal surface sites. It is also found that the introduction of defects can change the electronic structure, magnetic properties, and chemical reactivity of 2D layered nanomaterials, and thus increase their sensitivity and selectivity [30,34]. DFT calculations play an important role in the studies on the influence and applications of such defects.

Sulfur vacancies (SVs) are among the most common defects in 2D transition-metal chalcogenides (TMDs). Li et al. [35] studied the effects of SVs on the adsorption behavior of gas molecules on monolayer MoS_2_ using first-principle calculations, including CO_2_, N_2_, H_2_O, CO, NH_3_, NO, O_2_, H_2_, and NO_2_. Among these gas species, CO_2_, N_2_, and H_2_O remain physically adsorbed on the surface of defective MoS_2_. The adsorption energy calculations and charge transfer analysis indicate that the defective MoS_2_ is more sensitive than pristine MoS_2_. The S vacancy can improve the performance of the MoS_2_ based gas sensors with a moderate recovery time. Furthermore, CO, NO, NH_3_, and O_2_ could even be chemisorbed at the S vacancy sites, leading to a more stable adsorption and larger changes in electronic properties of MoS_2_. Xiong et al. [29] and Qin et al. [36] demonstrated that S vacancies were the key factor for a high sensitivity of 2D SnS_2_ to NH_3_.

Because of the excellent activity exhibited by those defects, the sensing performance of materials can be enhanced via controlled synthesis and defect engineering. The atomic scale study of the structural defects by first-principle calculations brings up new opportunities. Zhou et al. [37] studied the intrinsic structural defects in monolayer MoS_2_, grown by the chemical vapor phase method, using the direct atomic resolution imaging and first-principle calculation methods. The atomic-resolution scanning transmission electron microscope annular dark field (STM-ADF) images clearly showed the structure of MoS_2_ and its defects, including the point defects, 60°/small-angle grain boundaries, and reconstructed edges. Furthermore, the similar defect structure was built and explored using DFT calculations. The structure stability of different kinds of defects was explored through their formation energies, and the effects of the observed defects on the electronic properties of MoS_2_ were also studied there. Thus, new insights were provided into how the growth conditions, structural defects, and material properties affect each other. Another discovery reported by Hong et al. [38] was that antisite defects with Mo replacing S were dominant point defects in physical vapor deposition (PVD)-grown MoS_2_, while the S vacancies dominated in the chemical vapor deposition (CVD) specimens. Further explorations into the growth mechanisms and defect formation energies calculations confirmed these experimental observations. Recently, Komsa et al. [39] suggested a new way of engineering the electronic structure of TMDs. Firstly, the corresponding electron energies required to produce the defects can be estimated on the basis of the displacement threshold energies (*Td*), which can be calculated by means of the DFT method. The required vacancies can then be created under exposure to corresponding electron beams. Finally, the vacancies created by the electron beam can be filled with impurity atoms to achieve doping in TMDs. In addition, the method of low-energy argon ion beam sputtering can also selectively desulfurize the monolayer MoS_2_ without significantly depleting the molybdenum [40].

Doping is one of the most frequently used methods to modify the performance of materials. It has been reported that the doping of both nonmetal [41,42] (B, N, P, Cl, etc.) and transition-metal [43,44,45,46,47] (Cu, Ag, Au, Pd, Pt, Ni, Nb, etc.) elements can change their electronic and magnetic properties, as well as the chemical activity of 2D nanomaterials, as shown in Table 2. According to theoretical calculations, dopants can always occupy the vacancies because of their large binding energies with surrounding atoms [48]. Additionally, the electronic properties of substrates could be modulated, even undergoing transitions from semiconductor to half-metal behavior [34], due to the presence of the dopants. Furthermore, the strong orbital hybridization between dopants and gas molecules can significantly improve the gas sensitivity and can promote the transfer of electrons [49]. In particular, the partially occupied orbitals of the dopant atoms near the Fermi level play a crucial role [47,50]. Calculations of the optimized geometry structure, adsorption energy, density of states, and charge transfer analysis indicate that most of the gas molecules settle down on the doped substrates by chemisorption rather than by weak physisorption, which dominates on the pristine surface. For instance, Xin et al. [51] found that the adsorption energies of H_2_CO molecules on the surface of Ti-doped and V-doped graphene were about one order of magnitude higher than those on the pristine graphene. The charge transfer between the H_2_CO and Ti-doped graphene also showed a 38-times improvement compared with pristine graphene. When NO was adsorbed on B- and P- doped monolayer MoS_2_, the band gap demonstrated a decrease of 0.52 eV and 0.69 eV, respectively. leading to an improvement of the electrical conductivity [41]. Zhang et al. [52] systematically investigated Ni-, Fe-, and Co-doped MoS_2_ film sensors at room-temperature through experiments and by means of DFT calculations. The results showed that the Ni-doped MoS_2_ film sensors exhibited a relatively short response and recovery time, and an excellent stability towards SO_2_ gas.

Grain boundaries are important defects in materials. The disordered atomic arrangement at the grain boundaries makes their properties different from those of the grain interior. Previous experiments have revealed the influence of grain boundaries on the properties of sensing materials [56,57]. Choi et al. [46] doped Nb into two-dimensional MoS_2_ layered nanomaterials in order to improve their gas sensing properties. The ADF-STEM images clearly show that a decrease in the grain size correlates with an increased concentration of Nb doping. Thus, a large number of grain boundaries and stable dopant Nb dopants were responsible for the improvement of the sensitivity and stability of the gas sensor. Furthermore, the Nb atoms can directly form chemical bonds with some gas molecules, in addition to indirectly enhancing the sensitivity of MoS_2_ through modulating their electronic properties by donating electrons or holes to the conduction or valence bands, respectively. The other calculation of gas adsorption on the Nb doped graphene sheets (Nb/G) was reported by Kumar et al. [54]. In their study, CO and SO_2_ molecules were demonstrated to be chemisorbed on the surface of Nb/G.

**Figure 4:**
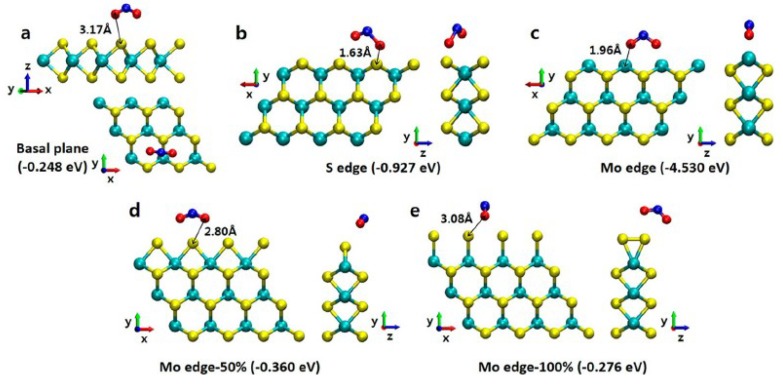
Schematics of NO_2_ molecules adsorbed on (**a**) the basal plane, (**b**) S edge, (**c**) Mo edge, (**d**) Mo edge-50%, (**e**) and Mo-edge-100%. Cyan, red, yellow, and blue spheres represent molybdenum, oxygen, sulfur, and nitrogen, respectively (reproduced from [60], with permission from American Chemical Society, 2018).

Edge sites always possess a high surface energy and thus are hard to form on the outside surfaces [58]. Edges, nevertheless, have more active sites than basal planes, because of their increased number of dangling bonds, maximal roughness, and different local stoichiometry [59]. Thus, it has been found that edge sites show much better surface properties for catalysis and gas-sensing performance. Cho et al. [60] verified through DFT calculations that the NO_2_ adsorption energies were higher on the edge sites than on the basal plane of MoS_2_. In particular, it was found that, because of the higher adsorption energy on the edge sites, the NO_2_ adsorption directly correlates with the density of the exposed edge sites on the MoS_2_ (see Figure 4). In this work, two different types of edge configurations with different coverages of S (50% and 100%) on the Mo edge were considered. Similarly, the ratio of the edge–surface sites has influence in the gas response and recovery of MoSe_2_ nanosheets [61]. 

Maximally exposing the edges on the film surfaces is a favorable way to enhance the sensitivity of the 2D layered nanomaterials. Forming active edge sites, which are thermodynamically unfavorable compared to the inert basal plane sites, presents a particular challenge. A method for the rapid sulfurization/selenization of MoS_2_ and MoSe_2_ thin films with vertically aligned layers was presented by Kong et al. [62]. With elemental sulfur/selenium powders as the precursors, e-beam evaporated ultrathin Mo films were converted into MoS_2_/MoSe_2_ films by a rapid sulfurization/selenization process in a horizontal tube furnace. Cho et al. [60] successfully synthesized horizontally aligned (basal-plane exposed) and vertically aligned (edge-exposed) MoS_2_ films using a rapid CVD sulfurization process. Under identical concentrations of the gas molecules, it was verified that gas sensors based on vertically aligned MoS_2_ films showed about a five-fold enhanced sensitivity to NO_2_ gas molecules compared to the horizontally aligned ones. Kibsgaard et al. [58] synthesized the continuous large-area thin films of a highly ordered double-gyroid MoS_2_ bicontinuous network with nanoscale pores so as to engender more exposed edge sites. 

Where strong binding to dopants and defect sites is advantageous for adsorption, it is disadvantageous for desorption, as it results in long recovery times in gas sensors. Additionally, vacancies and edges often become unstable upon the adsorption of O_2_ and H_2_O in air. Therefore, it is necessary to take these impurities into consideration during the simulation of the adsorption process.

### 4.2. Heterojunctions

In parallel with the study of the isolation of 2D nanomaterials, in-plane heterojunctions based on two different kinds of 2D nanomaterials, such as reduced graphene oxide (rGO)/oxide [63,64,65,66,67], TMDs/oxide [68,69,70], rGO/TMDs [71,72], and TMDs/TMDs [73], have received much attention. Such heterojunctions have been demonstrated to yield gas sensors that greatly enhanced the sensitivity. Knowledge about the gas sensing mechanisms of heterojunction materials is still limited, and the standards for choosing materials containing heterojunctions are not yet established. In general, when two kinds of materials with different work functions make up a heterojunction, the electrons at the lower work function side will flow across the interface to the higher work function side, leading to electronic depletion and enrichment areas, respectively [74]. A potential barrier will develop at the interface along with a p–n heterojunction. Under these specific circumstances, the barrier will be changed by the adsorption or desorption of gas molecules, resulting in an additional modulation of the total resistance of the gas sensor [63]. While the electronic enrichment side will attract more oxidizing gases, causing a higher change in the total resistance of the gas sensor [75], the reducing gas species will adsorb on the electronic depletion side, thus dramatically reducing the depletion region and hence causing large changes in the current flow [76]. 

With regard to materials containing heterojunctions, there are different explanations for the enhanced response. The work reported so far can be largely sub-divided into the following three categories: (i) graphene and its derivatives, reduced graphene oxides (rGO), and graphene oxides (GO); (ii) transition metal chalcogenides (TMDs); and (iii) metal oxides. Graphene has significant advantages of a large surface, high carrier mobility, and low noise [77]. In comparison, rGO and GO have rich functional groups and rGO has excellent semiconducting properties [78,79,80]. TMDs possess lots of active sites (sulfur vacancies, edges, and defects), semiconducting behavior, and a large surface and room temperature operability [72]. Conventionally-studied metal oxides have a high gas sensitivity at an elevated temperature because of chemical adsorption [16,81]. Therefore, it is a feasible strategy to improve gas-sensing properties of sensors by forming heterojunctions using two different kinds of 2D nanomaterials, or by fully exploiting the specific advantages of the sensing materials on each side.

Similarly, the properties of heterojunctions consisting of two kinds of 2D nanomaterials stitched together also can be studied using DFT calculations. A typical example is the investigation of the gas adsorption on MoS_2_/WS_2_ in-plane heterojunctions. The model of the in-plane MoS_2_/WS_2_ heterojunction was built and the adsorption behaviors of CO, H_2_O, NH_3_, and NO_2_ gas molecules on the heterojunction were investigated by means of DFT calculations by Sun et al. [73]. The results show that NO_2_ possesses the largest adsorption energy among all of the gas molecules considered, which was attributed to the changes in the heterojunction electronic structure that occurred upon charge transfer during adsorption. Furthermore, previous reports of first-principle calculations showed that the narrow band gaps of heterojunctions can induce a high density of charge and chemisorbed oxygen, so that the response of the SnO_2_/SnS_2_ hybrids becomes dramatically increased [82]. However, there are almost no theoretical calculation studies that compare the electronic structures, the binding energies, levels of charge transfer, adsorption behaviors, and geometrical structure of heterojunctions with their component materials. This may be due to the complexity of the respective model constructions.

### 4.3. Electrical Fileds

**Figure 5:**
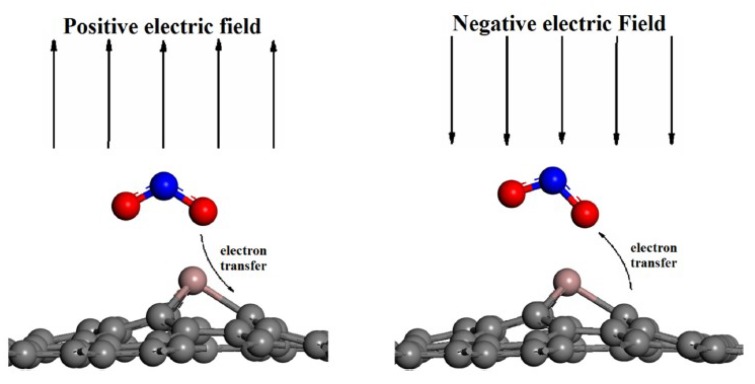
Schematic of the electron transfer mechanism under different electric fields (reproduced from [85], with permission from ELSEVIER, 2018).

**Figure 6:**
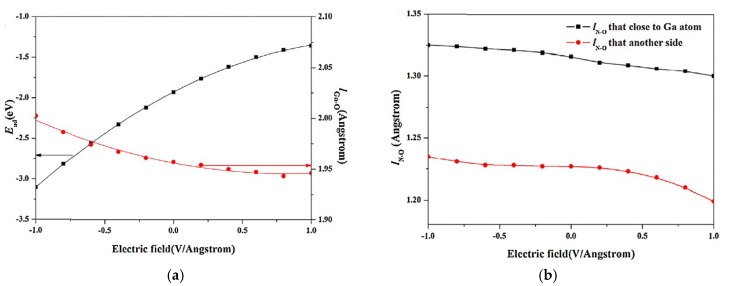
Variation of (**a**) adsorption energy (*E_ad_*) and bond length of Ga–O (*l_Ga–O_*), (**b**) charge transfer on NO_2_ molecule under various electric field (reproduced from [85], with permission from ELSEVIER, 2018).

It is well known that the charge transfer between gas molecules and 2D gas-sensing nanomaterials plays a crucial role in determining their properties as gas sensors. Electrical fieleds applied to the gas sensing materials can modulate their sensitivity and the reversibility of gas sensors [83]. A well-investigated example is CO adsorption on Al-doped graphene [84]. The DFT calculations show that a negative electric field, *F*, can increase the adsorption energy of CO on Al-doped graphene, while a positive F can weaken the adsorption. Furthermore, the CO molecules are desorbed from the graphene layer when F≥0.03 a.u., which is beneficial for the reversibility of the gas adsorption/desorption process. The advantage of the external electric field modulation of the gas sensitivity has also been demonstrated in the case of the adsorption/desorption processes on the surface of Ga-doped graphene [85]. The influence of the electric fields was attributed to the polarization of the charge density and charge particle [86,87,88]. The electron transfer mechanism between NO_2_ and the Ga-doped graphene monolayer under different electric fields is shown in Figure 5 and Figure 6. The charge transferred from NO_2_ toward Ga-doped graphene is associated with a movement of the gas molecules towards the monolayer under a positive electric field, while a completely opposite behavior is observed under the negative electric fields. According to the charge analysis, there was an additional electron transfer of about 0.26 electrons from the Ga-doped graphene to the NO_2_ gas molecules under at an electric field strength of −1 V/Angstrom, as compared to the no-field case, as shown in Figure 6b. 

## 5. Conclusion and Outlook

A variety of investigations on the gas sensing properties of two-dimensional nanomaterials have been carried out. Theoretical calculations play a crucial role in both elucidating the gas sensing mechanisms of 2D layered nanomaterials, as well as for enhancing the gas-sensing performance of sensors based on 2D layered nanomaterials. Various models can be established to simulate the gas adsorption process and to calculate adsorption behaviors, including the adsorption energy, distance between gas molecules and sensing materials, geometrical structure, and charge transfer analysis to judge the sensitivity of the materials. One can also calculate the changes in the electronic properties of the sensing materials that result from the gas adsorption, which is relevant for assessing the nature of the gas sensing mechanisms. However, there are also some limitations to this theoretical approach. For instance, the actual situations are always more complicated than those situations conceived in the ideal models. Presently, some conditions cannot be simulated, such as external light irradiation.

In the future, there will be great opportunities for 2D nanomaterials in the field of advanced gas sensors. In addition to defect functionalization, heterojunctions, and external electric field modulation, light irradiation [89] will significantly improve the gas sensing properties of 2D nanomaterials. It is expected that the combination of several methods may be the most effective to improve on the sensitivity of the gas sensors. A promising example is the adsorption of NO_2_ on MoS_2_ nanosheet/ZnO nanowire heterojunctions, which exhibits ultra-sensitivity under UV light [90].

## Figures and Tables

**Table 1:** Adsorption of several common gas molecules on 2D nanomaterials: Adsorption energy (*Ea*), charge transfer (∆*Q*), and distance between gas molecules and materials (*d*).

Materials	Gas Molecules	*Ea*/meV	∆*Q*/e	*d*/Å	Method	Reference
Graphene	H_2_O	−47	0.025	3.5	ABINIT ^1^ code/GGA ^2^	[21]
	NH_3_	−31	−0.027	3.81		
	CO	−14	−0.013	3.74		
	NO	−29	−0.017	3.76		
	NO_2_	−67	0.099	3.61		
WS_2_	NH_3_	−216	−0.227	2.49	VASP ^3^/LDA ^4^	[31]
	H_2_O	−229	0.081	2.63		
WS_2_	NH_3_	−630	-	2.25	SIESTA ^5^//LDA + DFT-D2 ^6^	[23]
	NO_2_	−1520	-	2.39		
	NO	−880	-	2.6		
	O_2_	−430	-	2.03		
WSe_2_	NH_3_	−560	-	2.37		
	NO_2_	−1320	-	2.3		
	NO	−1050	-	2.62		
	O_2_	−440	-	2.1		
WSe_2_	O_2_	−8.7	0.0182	3.21	VASP/GGA-PBE ^7^	[32]
	CO	−9.2	0.0089	3.76		
	NH_3_	−42	−0.0172	3.11		
	H_2_O	−45	0.0186	2.78		
	NO	−25	0.0346	2.95		
	NO_2_	−67	0.1165	3.04		
MoTe_2_	SO_2_	−245	0.086	3.437	VASP/GGA-PBE + vdW ^8^ correction	[33]
	H_2_S	−212	0.017	3.662		
	NH_3_	−235	0.069	3.453		
SnS_2_	CH_4_	−182	-	-	CRYSTAL14 ^9^/B3LYP ^10^	[18]
	CO_2_	−191	-	-		
	H_2_	−53	-	-		
	H_2_S	−199	-	-		
	NH_3_	−215	-	-		
	NO_2_	−367	0.048	2.41		
	O_2_	1430	-	-		

^1^ ABINIT is a software suite used to calculate the optical, mechanical, vibrational, and other observable properties of materials. ^2^ GGA is the generalized gradient approximation. ^3^ VASP is the Vienna Ab initio Simulation Package. ^4^ LDA: the local density approximation. ^5^ SIESTA is Spanish Initiative for Electronic Simulations with Thousands of Atoms package. ^6^ DFT-D2 is a kind of van der Waals correction method. ^7^ PBE is the Perdew–Burke–Ernzerhof. ^8^ vdW is the van der Waals. ^9^ CRYSTAL14 is the Gaussian basis set ab initio package. ^10^ B3LYP is a kind of hybrid exchange-correlation functional.

**Table 2:** Influence of dopant species on the 2D nanomaterials for gas sensing: First-principle studies.

Substrate	Elements	Gas Species	Mechanism	Reference
Graphene	Ti, V	H_2_CO	*Ea* increased by one order of magnitude	[51]
Graphene	B, N, P, Al	CH_4_	Physical adsorption	[53]
Graphene	B, N	CO, NO, NO_2_, NH_3_	*Ea* of NO and NO_2_ enhanced by N-and B-doping	[26]
Graphene	Nb	CO, NH_3_, CH_4_, SO_2_, H_2_S	Formed chemical bonds	[54]
MoS_2_	Cl, P, Si	H_2_CO	P and Si provided p-type doping	[49]
MoS_2_	Al, Si, P	NO_2_, NH_3_	Charge transfer between dopant and gas molecules increased by orbital hybridization	[49]
MoS_2_	Co, Ni, Rh, Ru, Pd, Ir, Pt, Au	O_2_	Partially occupied d orbital of TMs ^1^ play crucial role	[47]
MoS_2_	Fe, Co, Ni, Cu, Ag, Au, Rh, Pd, Pt, Ir	NO, CO, O_2_, NH_3_	Fe and Co possess best adsorption ability, thermal stability and chemical activity	[45]
MoS_2_	V, Cr, Mn, Fe, Co, H, B, N, F	-	Conductivity and magnetic properties changed	[34]
MoS_2_	V, Nb, Ta	CO, NO, H_2_O, NH_3_	Orbital overlap between n, d orbitals of metal, and gas molecules	[50]
MoS_2_	B, Cl, P	NO	Decreased band gap, larger charge transfer, and higher adsorption energy after gas adsorption	[41]
MoS_2_	Ni, Fe, Co	SO_2_	Improved response, recovery properties, and stability after Ni doping	[52]
MoS_2_	Pt, Au	SO_2_, SOF_2_, SO_2_F_2_	Enhanced sensitivity to SO_2_	[55]
MoS_2_	Au, Ag, Pt, Pd, Sc, Y	H_2_	Efficiency of doping is related to work function of dopants	[34]
MoS_2_	Cu	NO, NO_2_, O_2_, NH_3_	Related to charge transfer and orbital hybridization between Cu and gas molecules	[45]
MoSe_2_	Nb	NO_2_	Increased the density of grain boundaries	[46]
PtSe_2_	Ge, As, Br	H_2_, O_2_, CO, CO_2,_ NH_3_, NO, NO_2_	Charge transfer between gas molecules and empty 4p orbitals of Ge and As	[50]

^1^ TMs is the abbreviation for transition metals.
